# Supplementary material for: Measuring the shape of the biodiversity-disease relationship across systems reveals new findings and key gaps
Source: Nat Commun. 2019 Nov 6;10:5032. doi: 10.1038/s41467-019-13049-w (PMC6834853; doi:10.1038/s41467-019-13049-w)
Supplement: Supplementary file 1 — Supplementary Information [file 41467_2019_13049_MOESM1_ESM.pdf]

Supplementary Information for

Measuring the shape of the biodiversity-disease relationship across systems reveals new findings and key gaps

Fletcher W. Halliday, Jason R. Rohr

Supplementary Figures
Supplementary Tables
Supplementary Note 1. Supplemental analysis of the shape of biodiversity-disease relationships using the adjusted R^2 and Bayesian Information Criterion (BIC) for model selection
Supplementary Note 2. Supplemental analysis of spatial scale moderating the shape of the biodiversity-disease relationship
Supplementary References

Supplementary Figures

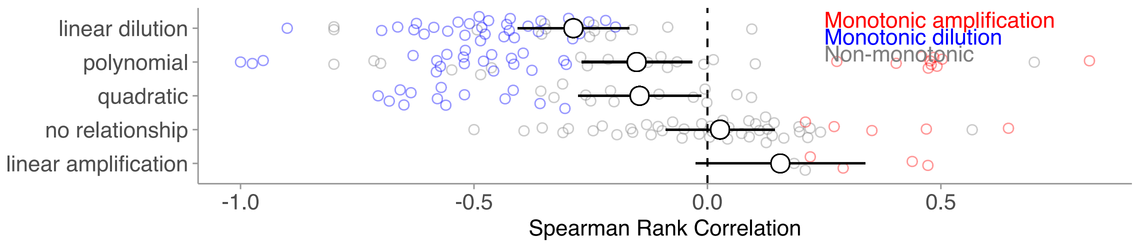

Supplementary Figure 1. Results of the analysis comparing the shape of the biodiversity-disease relationship quantified using model selection using AIC to the Spearman rank correlation. Points are model-estimated means and error bars are 95% confidence intervals. The colored points show the distribution of the raw data. Studies exhibiting monotonic amplification effects ($\rho>0$, Spearman $p<0.05$) are shown in red, monotonic dilution effects ($\rho<0$, Spearman $p<0.05$) are shown in blue, and non-significant or non-monotonic relationships are shown in grey. Source data are provided as a Source Data file.

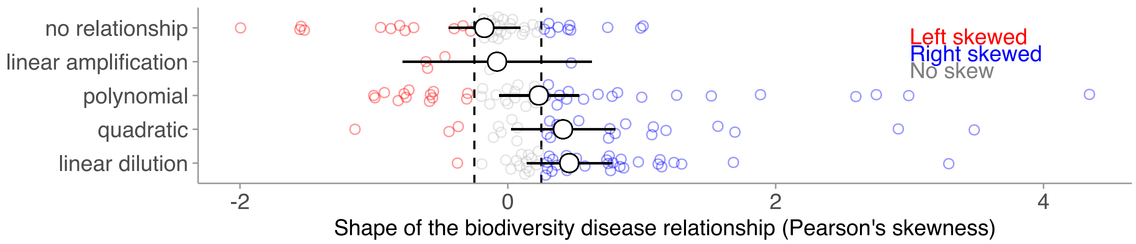

Supplementary Figure 2. Results of the analysis comparing the shape of the biodiversity-disease relationship quantified using model selection using AIC to Pearson's skewness. Points are model-estimated means and error bars are 95% confidence intervals. The colored points show the distribution of the raw data. Left-skewed relationships (Pearson's skewness<0.25) are shown in red, right-skewed relationships (Pearson's skewness>0.25) are shown in blue, and non-skewed relationships are shown in grey. Source data are provided as a Source Data file.

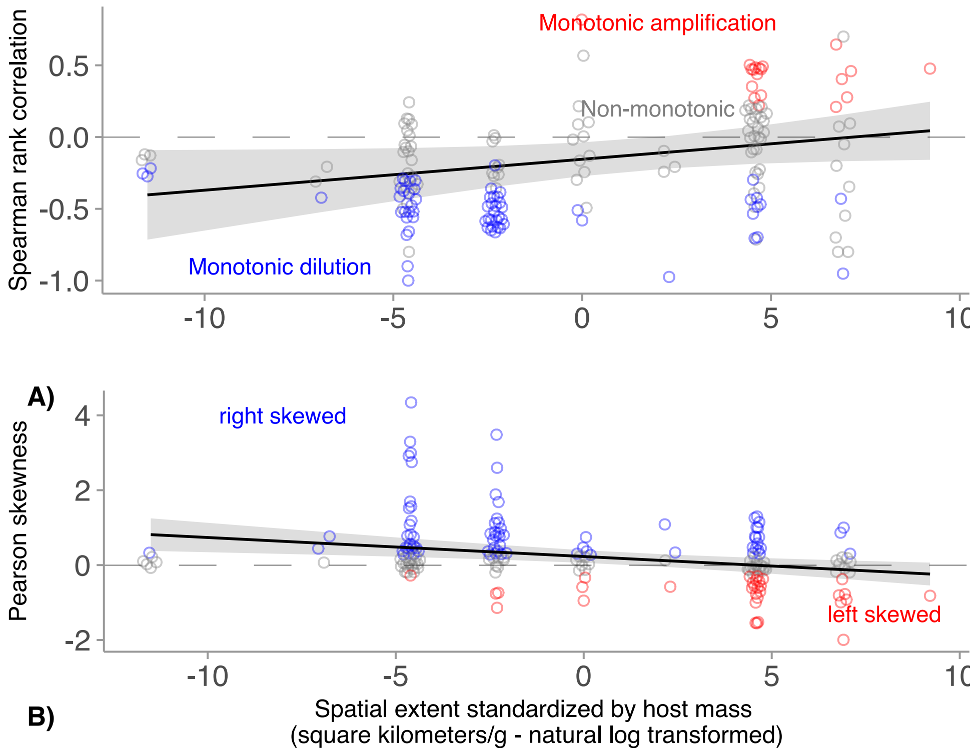

Supplementary Figure 3. Results of the analyses relating spatial scale to the shape of the biodiversity-disease relationship after standardizing spatial scale by dividing spatial scale by estimated host biomass. Points represent each published biodiversity-disease relationship, colored by their estimated shape (red = Monotonic amplification in panel A and left-skewed in panel B; blue = Monotonic dilution in panel A and right-skewed in panel B; grey = non-significant or non-monotonic in panel A non-skewed in panel B). Solid lines indicate the estimated fit of a multilevel random effects model, and grey ribbons indicate the 95% confidence intervals. Spatial scale moderates the relationship between biodiversity and disease even after standardizing spatial scale by host biomass: A) Spearman rank correlation between biodiversity and disease was positively associated with spatial extent, and B) Pearson's skewness was negatively associated with spatial extent. Source data are provided as a Source Data file.

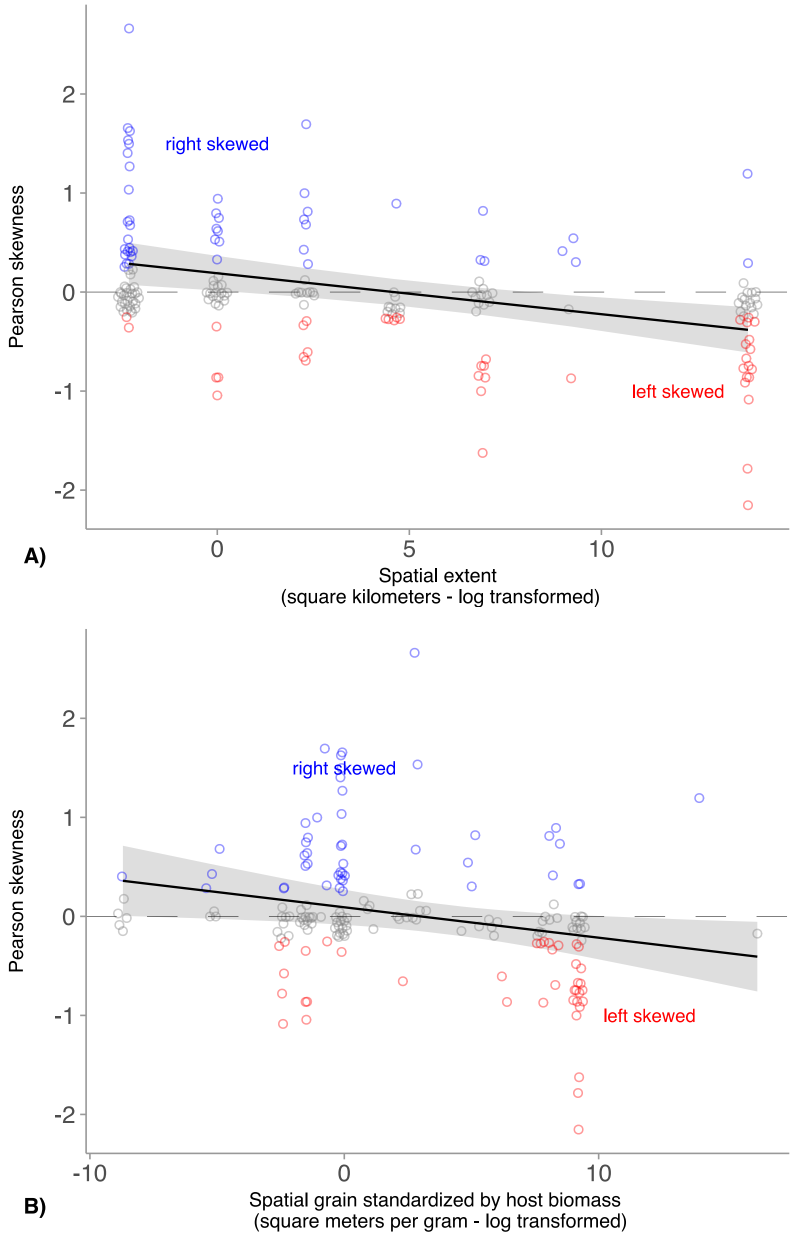

Supplementary Figure 4. Results of the analyses relating spatial scale to the shape of the biodiversity-disease relationship after constraining the curves to pass through the origin. Points represent each biodiversity-disease relationship, colored by their estimated shape (red = left-skewed, blue = right-skewed, grey = non-skewed). Solid lines indicate the estimated fit of a multilevel random effects model, and grey ribbons indicate the 95% confidence intervals. Spatial scale moderates the relationship between biodiversity and disease even after constraining the curves to pass through the origin. Specifically, Pearson's skewness between biodiversity and disease was negatively associated with A) spatial extent (Type III ANOVA p=0.0005) and B) spatial grain standardized by host biomass (Type III ANOVA p=0.020). Source data are provided as a Source Data file.

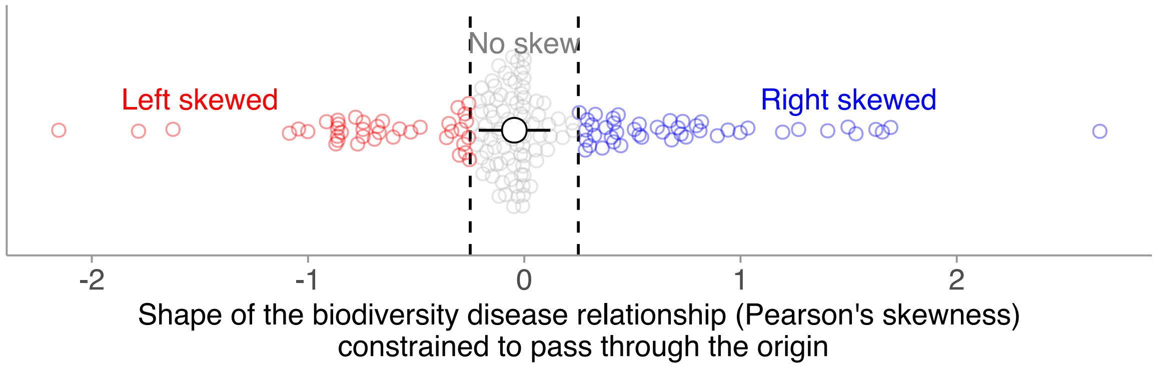

Supplementary Figure 5. Results of the analysis assessing average Pearson’s skewness across all studies after constraining the curves to pass through the origin. The black point is the model-estimated mean and error bars are 95% confidence intervals. The colored points show the distribution of the raw data. Left-skewed relationships (Pearson’s skewness<0.25) are shown in red, right-skewed relationships (Pearson’s skewness>0.25) are shown in blue, and non-skewed relationships are shown in grey. Even though constraining curves to fit through the origin shifted the estimated skew, on average, the constrained curves were not significantly left-skewed (T-test $p = 0.55$). Source data are provided as a Source Data file.

Supplementary Tables

Supplementary Table 1. Models testing whether the effect of spatial scale on biodiversity-disease relationships depends on disease or diversity metric

	A) Spearman correlation coefficient			B) Pearson's skewness		
	**DF**	**F-value**	**p-value**	**DF**	**F-value**	**p-value**
Spatial extent	39.7	0.20	0.660	165	0.23	0.629
Diversity metric	42.1	0.73	0.489	165	1.28	0.282
Disease metric	35.2	0.54	0.660	165	1.72	0.166
Spatial extent × Diversity metric	41.4	0.29	0.752	165	0.71	0.492
Spatial extent × Disease metric	32.3	0.66	0.522	165	1.83	0.164

Type III Analysis of Variance Table with Satterthwaite's method

DF: Denominator degrees of freedom

Supplementary Table 2. Models of ecological factors moderating the effect of spatial scale on biodiversity-disease relationships that have been constrained to pass through the origin.

	DF	F-value	p-value
Spatial extent	27.6	0.231	0.635
Human	54.0	0.554	0.460
Route	53.4	0.167	0.685
Macroparasite	19.4	0.195	0.664
Manipulative	43.4	0.909	0.346
Spatial extent × Human	34.8	0.037	0.849
Spatial extent × Route	48.8	0.243	0.624
Spatial extent × Macroparasite	23.3	0.916	0.348
Spatial extent × Manipulative	29.9	0.019	0.892
Spatial grain	35.7	0.346	0.560
Human	37.9	0.684	0.413
Route	31.8	0.055	0.816
Macroparasite	36.1	0.425	0.518
Manipulative	27.3	0.047	0.830
Spatial grain × Human	35.0	0.142	0.708
Spatial grain × Route	34.5	1.170	0.287
Spatial grain × Macroparasite	36.8	0.183	0.671
Spatial grain × Manipulative	32.6	0.372	0.546

Type III Analysis of Variance Table with Satterthwaite's method
DF: Denominator degrees of freedom

Supplementary Note 1. Supplemental analysis of the shape of biodiversity-disease relationships using the adjusted R^2 and Bayesian Information Criterion (BIC) for model selection

We tested whether the shape of biodiversity-disease relationships depended on the model-selection criterion by performing the same analysis of the shape of biodiversity-disease relationships using the adjusted R^2 statistic and BIC for model selection. In the analysis using AIC, out of the 205 studies that included more than three levels of biodiversity, 67% were best fit by a linear, second-order, or third-order polynomial model (i.e., exhibited a relationship between biodiversity and disease). Of these studies, biodiversity-disease relationships were commonly non-linear, as predicted. More specifically, 61% exhibited non-linear relationships (either second- or third-order polynomial), while 6% exhibited a linear, positive biodiversity-disease relationship (e.g., linear amplification effect), and 33% exhibited a linear, negative biodiversity-disease relationship (e.g., linear dilution effect). Whether the best-fitting model was linear, nonlinear, or intercept-only did not depend on the number of diversity levels per study (Supplementary Table 1).

Using adjusted R^2 for model selection, out of the 205 studies that included more than three levels of biodiversity, 80% were best fit by a linear, second-order, or third-order polynomial model (i.e., exhibited relationship between biodiversity and disease). Of these studies, biodiversity-disease relationships were still most commonly non-linear, as predicted. More specifically, 71% exhibited non-linear relationships (either second- or third-order polynomial fits), while 5% exhibited a linear, positive biodiversity-disease relationship (e.g., linear amplification effect), and 24% exhibited a linear, negative biodiversity-disease relationship (e.g., linear dilution effect). Whether the best fitting model was linear, nonlinear, or intercept-only did not depend on the number of diversity levels per study (Supplementary Table 1).

Using BIC for model selection, out of the 205 studies that included more than three levels of biodiversity, 49% were best fit by a linear, second-order, or third-order polynomial model (i.e., exhibited a relationship between biodiversity and disease). Of these studies, biodiversity-disease relationships were still most commonly non-linear, as predicted. More specifically, 53% exhibited non-linear relationships (either second- or third-order polynomial), while 6% exhibited a linear, positive biodiversity-disease relationship (e.g., linear amplification effect), and 41% exhibited a linear, negative biodiversity-disease relationship (e.g., linear dilution effect). Whether the best-fitting model was linear, nonlinear, or intercept-only did not depend on the number of diversity levels per study (Supplementary Table 1).

Next, we tested whether the relationship between biodiversity and disease was monotonic and positive (disease increases, but may level off, as diversity increases), monotonic and negative (disease decreases but may level off as diversity increases), or non-monotonic (disease increases with diversity at low levels, but eventually decreases at high enough diversity). We quantified the shape of each biodiversity-disease relationship using Spearman rank correlations. Non-monotonic biodiversity-disease relationships can exhibit both amplification and dilution, and the shape of non-monotonic biodiversity-disease relationships might influence the conditions under which amplification and dilution are expected to occur. When biodiversity-disease relationships are left skewed, this might suggest that these systems experience more amplification than dilution, because the majority of diversity levels and systems occur where the relationship between biodiversity and disease risk is positive. In contrast, when the shape is right skewed, then dilution occurs over a wider range of diversity levels, increasing the chances that systems experience dilution. We therefore also assessed the skew of each biodiversity-disease relationship by fitting an unconstrained curve to each biodiversity-disease relationship, and then calculating Pearson's skewness on the shape of that curve.

We verified that studies exhibiting monotonic dilution, monotonic amplification, and non-monotonic relationships (categorized using the Spearman rank correlation) predicted Pearson's

skewness. As expected, studies exhibiting monotonic dilution effects were significantly right-skewed (T-test p<0.0001), and studies exhibiting monotonic amplification effects were significantly left-skewed (T-test p<0.0001). Studies exhibiting non-monotonic relationships were not significantly skewed (T-test p=0.80), indicating that non-monotonic biodiversity-disease relationships were equally likely to show amplification or dilution. These results were qualitatively similar for the analysis comparing intercept-only, linear, second-order, and third-order polynomial regression models with AIC as the selection criterion (Supplementary Figure 1, Supplementary Figure 2), for the analysis using adjusted R^2 as the selection criterion (Supplementary Figure 6, Supplementary Figure 7), and for the analysis using BIC as the selection criterion (Supplementary Figure 8, Supplementary Figure 9).

Finally, we compared the adjusted R^2 for the best model using each model selection criterion (Supplementary Figure 10). All models that were best fit by a linear, second-order, or third-order polynomial model (i.e., exhibited a relationship between biodiversity and disease) accounted for a considerable amount of variation, regardless of the model selection criterion that was used.

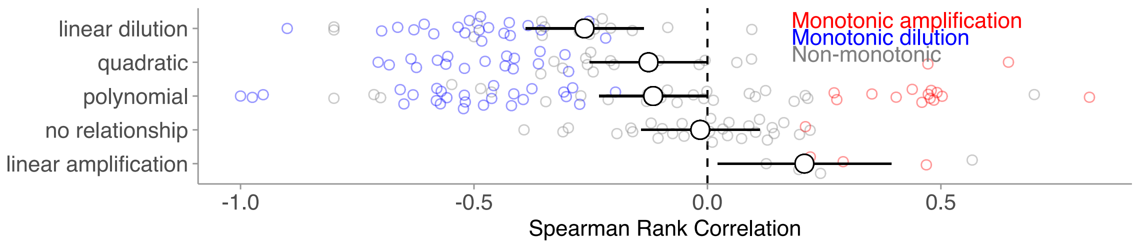

Supplementary Figure 6. Results of the analysis comparing the shape of the biodiversity-disease relationship quantified using model selection with adjusted R^2 to the Spearman rank correlation. Points are model-estimated means and error bars are 95% confidence intervals. The colored points show the distribution of the raw data. Studies exhibiting monotonic amplification effects (ρ>0, Spearman p<0.05) are shown in red, monotonic dilution effects (ρ <0, Spearman p<0.05) are shown in blue, and non-significant or non-monotonic relationships are shown in grey. Source data are provided as a Source Data file.

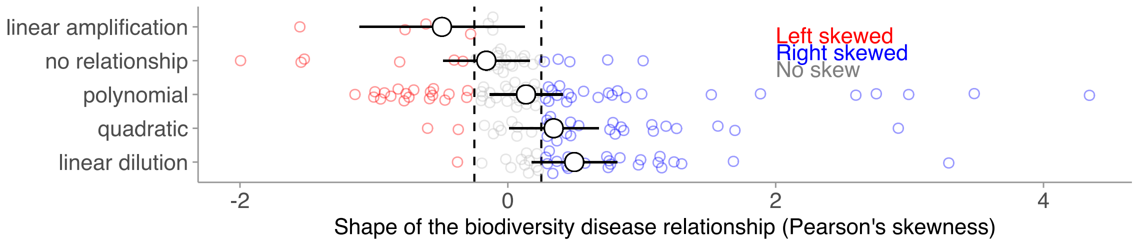

Supplementary Figure 7. Results of the analysis comparing the shape of the biodiversity-disease relationship quantified using model selection with adjusted R^2 to Pearson's skewness. Points are model-estimated means and error bars are 95% confidence intervals. The colored points show the distribution of the raw data. Left-skewed relationships (Pearson's skewness<0.25) are shown in red, right-skewed relationships (Pearson's skewness>0.25) are shown in blue, and non-skewed relationships are shown in grey. Source data are provided as a Source Data file.

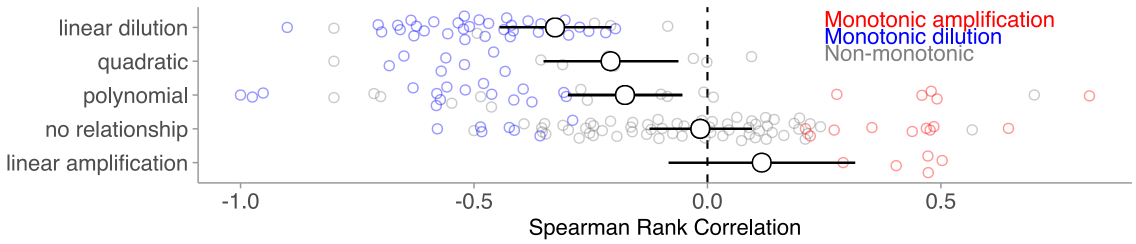

Supplementary Figure 8. Results of the analysis comparing the shape of the biodiversity-disease relationship quantified using model selection with BIC to the Spearman rank correlation. Points are model-estimated means and error bars are 95% confidence intervals. The colored points show the distribution of the raw data. Studies exhibiting monotonic amplification effects (ρ>0, Spearman p<0.05) are shown in red, monotonic dilution effects (ρ <0, Spearman p<0.05) are shown in blue, and non-significant or non-monotonic relationships are shown in grey. Source data are provided as a Source Data file.

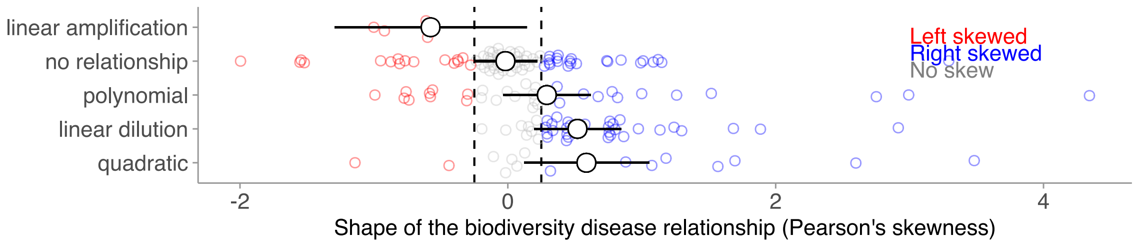

Supplementary Figure 9. Results of the analysis comparing the shape of the biodiversity-disease relationship quantified using model selection with BIC to Pearson's skewness. Points are model-estimated means and error bars are 95% confidence intervals. The colored points show the distribution of the raw data. Left-skewed relationships (Pearson's skewness<0.25) are shown in red, right-skewed relationships (Pearson's skewness>0.25) are shown in blue, and non-skewed relationships are shown in grey. Source data are provided as a Source Data file**.**

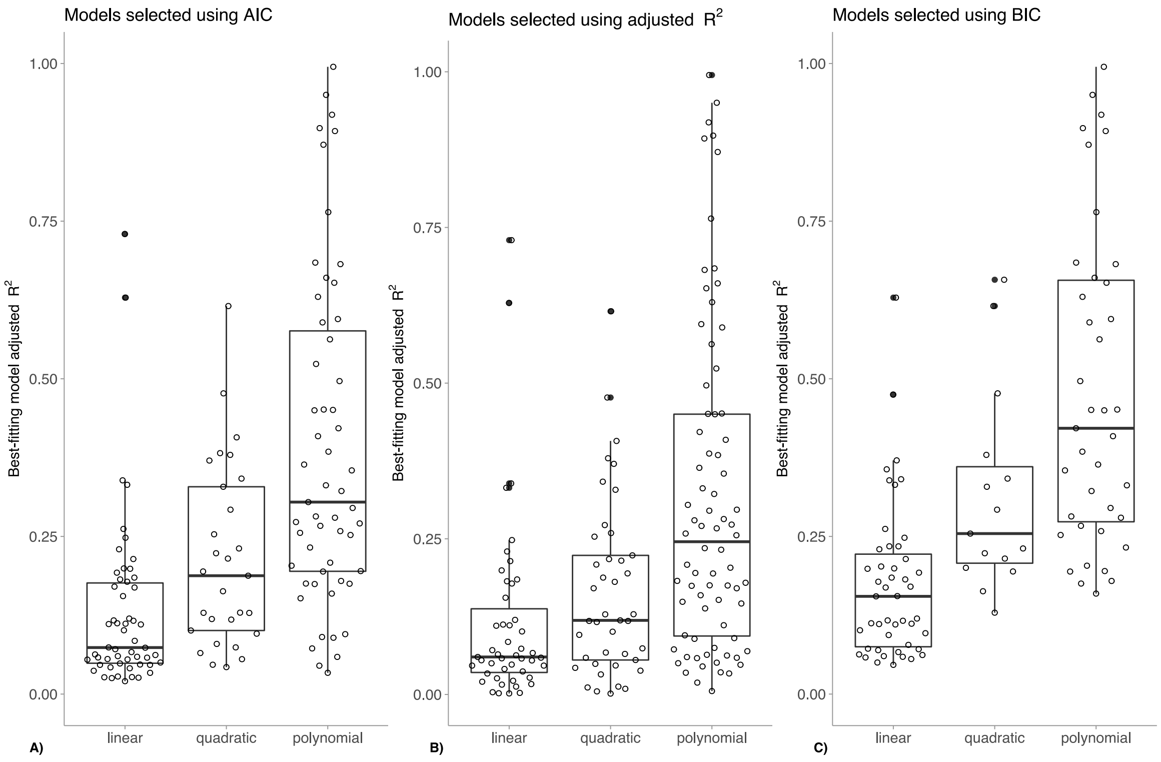

Supplementary Figure 10. Comparison of adjusted R^2 for the models that were best fit by a linear, second-order (quadratic), or third-order polynomial (polynomial) using three different model selection criteria. A) The best models selected using AIC. B) The best models selected using the adjusted R^2. C) The best models selected using BIC. The points show the distribution of the raw data. The box shows the first and third quartiles, the middle line shows the median, and the whiskers extend from the box to the largest and smallest values, no more than 1.5x the interquartile range. Source data are provided as a Source Data file**.**

Supplementary Note 2. Supplemental analysis of spatial scale moderating the shape of the biodiversity-disease relationship

We tested whether the shape of the biodiversity-disease relationship was moderated by spatial scale, measured both as the spatial extent and spatial grain of each study. Spatial extent represents the total area over which a study is conducted, including all measures of biodiversity and disease for a given study. Spatial grain represents the area over which a single biodiversity and disease estimate are collected. The effective spatial grain depends strongly on the size of a host (e.g., a redwood tree could not be studied in a 1m^2 plot, but a grass could). We therefore assessed spatial grain, standardized by host biomass.

We tested whether the Spearman rank correlation coefficient between biodiversity and disease or Pearson's skewness were influenced by spatial scale by fitting four separate models, each with one response (Rho or skew) and one predictor (extent or grain). Parasite species did not explain any residual variance in the models, leading to a computational singularity, and so this factor was omitted from the random effects in the models.

Spearman's rho was positively associated with spatial extent and grain. Incorporating the shape of non-monotonic relationships did not alter this result: Pearson's skewness was significantly associated with spatial extent and grain as well (Supplementary Figure 11).

We found significantly monotonic negative and right-skewed relationships occurring at spatial extents <100km^2 (roughly the size of a small city) and a spatial grains <25m^2g^{-1} (roughly the size of a small city for a typical human parasite). We found significantly monotonic positive and left-skewed relationships occurring in studies occupying >1,000,000km^2 (roughly the size of France and Spain combined), but we did not observe significantly monotonic positive and left-skewed relationships at any spatial grain.

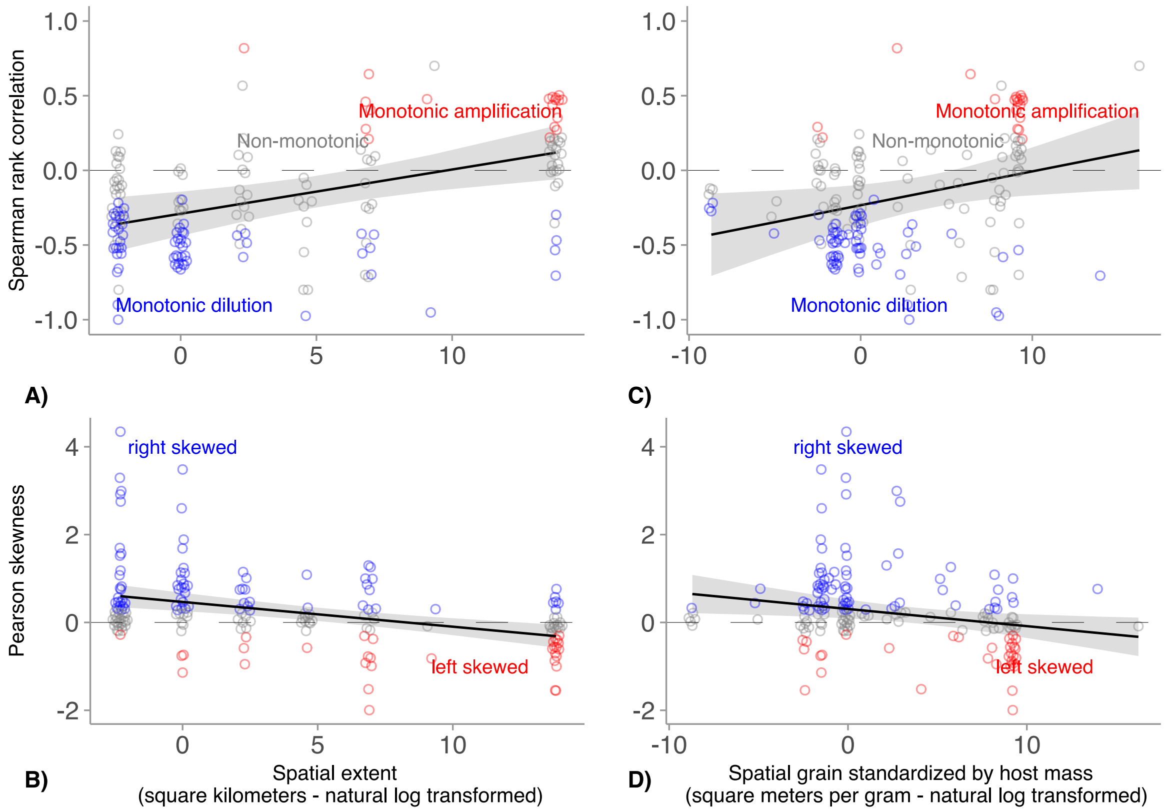

Supplementary Figure 11. Results of the analyses relating spatial scale to the shape of the biodiversity-disease relationship. Points represent each published biodiversity-disease relationship, colored by their estimated shape (red = Monotonic amplification in panels A and C and left-skewed in panels B and D; blue = Monotonic dilution in panels A and C and right-skewed in panels B and D; grey = non-significant or non-monotonic in panels A and C non-skewed in panels B and D). Solid lines indicate the estimated fit of a multilevel random effects model, and grey ribbons indicate the 95% confidence intervals. Spatial scale moderates the relationship between biodiversity and disease: A) Spearman rank correlation between biodiversity and disease was positively associated with spatial extent. B) Pearson's skewness was negatively associated with spatial extent. C) Spearman rank correlation between biodiversity and disease was positively associated with spatial grain standardized by host mass. D) Pearson's skewness was negatively associated with spatial grain standardized by host mass. Source data are provided as a Source Data file**.**

Importantly, only three papers included measurements at the scale of significant amplification, and one of these papers (Wood et al. 2017[1]), which contributed 22 out of 44 data points at the largest extent, used a distinct metric of disease, DALYs.

We tested whether the effect of spatial scale on the shape of biodiversity-disease relationships was sensitive to the study by Wood et al[1] by omitting this study and reanalyzing the data. Omitting this study did not remove the significant effect of spatial extent on the skewness of the biodiversity-disease relationship, but did eliminate the significant effect of spatial extent on the monotonicity and direction of the relationship and eliminated the effect of spatial grain on biodiversity-disease relationships (Supplementary Figure 12).

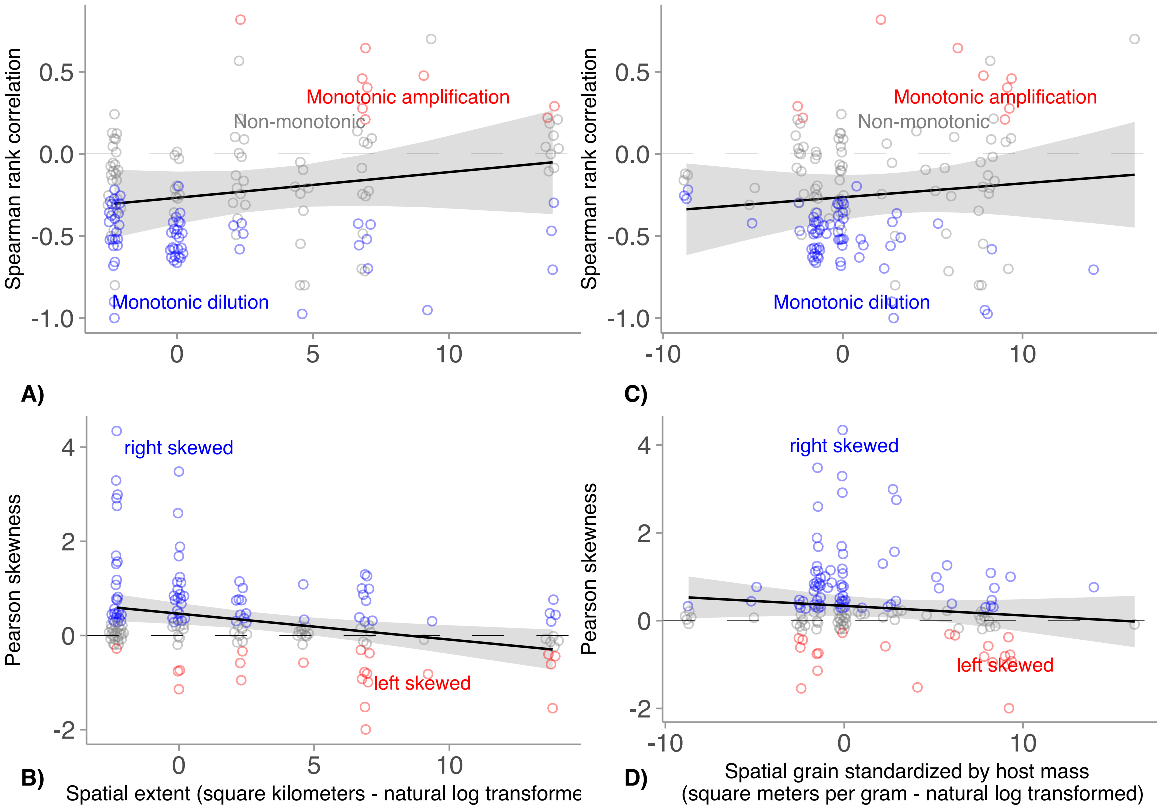

Supplementary Figure 12. Results of the analyses relating spatial scale to the shape of the biodiversity-disease relationship, omitting the study by Wood et al 2017[1]. Points represent each published biodiversity-disease relationship, colored by their estimated shape (red = Monotonic amplification in panels A and C and left-skewed in panels B and D; blue = Monotonic dilution in panels A and C and right-skewed in panels B and D; grey = non-significant or non-monotonic in panels A and C non-skewed in panels B and D). Solid lines indicate the estimated fit of a multilevel random effects model, and grey ribbons indicate the 95% confidence intervals. Even after removing the study by Wood et al 2017, spatial scale still moderates one relationship between biodiversity and disease: the relationship between spatial extent and Pearson's skewness. The remaining effects, although no longer significant, remain qualitatively similar. A) Spearman rank correlation between biodiversity and disease was not significantly associated with spatial extent. B) Pearson's skewness was negatively associated with spatial extent. C) Spearman rank correlation between biodiversity and disease was not significantly associated with spatial grain standardized by host mass. D) Pearson's skewness was not significantly associated with spatial grain standardized by host mass. Source data are provided as a Source Data file.

We found qualitatively similar results if we grouped all of the samples from Wood et al[1] in the random effects of the models. Treating all parasites and years of the Wood et al study as non-independent did not remove the significant effect of spatial extent on the skewness of the biodiversity-disease relationship, but did eliminate the significant effect of spatial extent on the monotonicity and direction of the relationship and eliminated the effect of spatial grain on all tested biodiversity-disease relationships (Supplementary Figure 13).

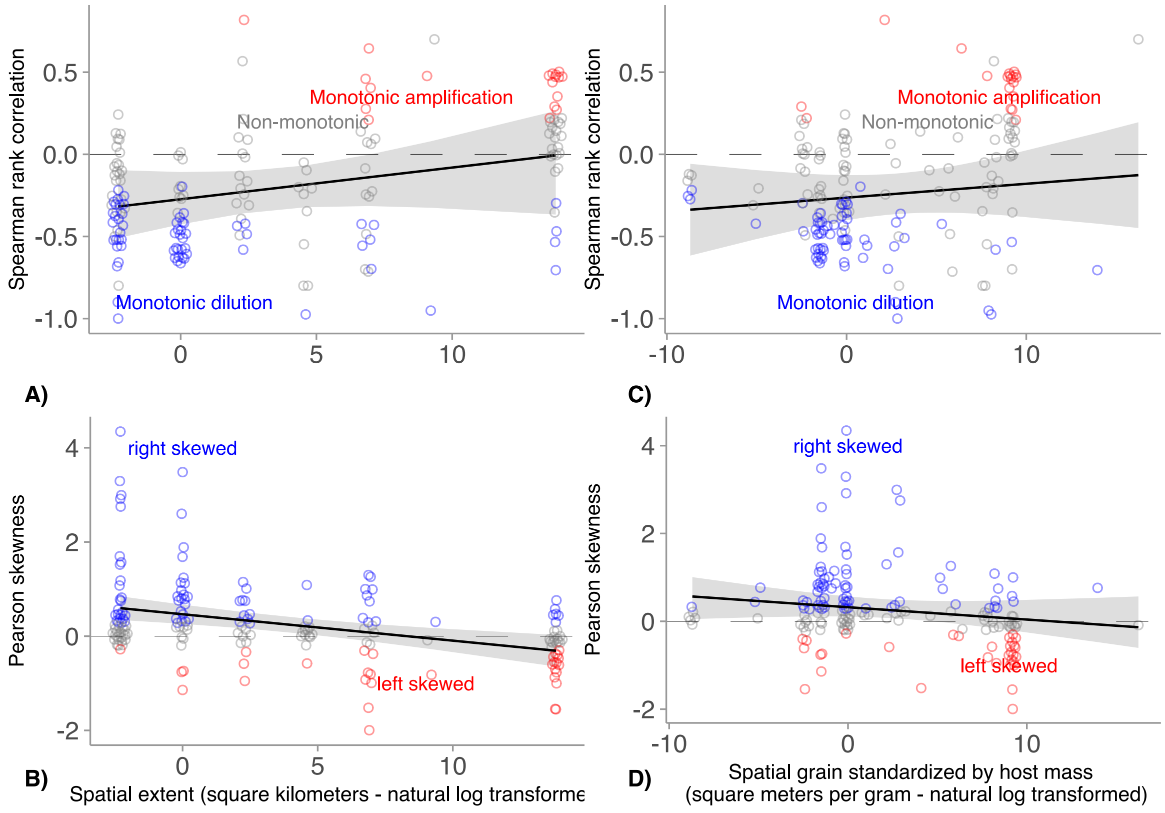

Supplementary Figure 13. Results of the analyses relating spatial scale to the shape of the biodiversity-disease relationship, treating all parasites and years of the study by Wood et al 2017[1] as non-independent. Points represent each published biodiversity-disease relationship, colored by their estimated shape (red = Monotonic amplification in panels A and C and left-skewed in panels B and D; blue = Monotonic dilution in panels A and C and right-skewed in panels B and D; grey = non-significant or non-monotonic in panels A and C non-skewed in panels B and D). Solid lines indicate the estimated fit of a multilevel random effects model, and grey ribbons indicate the 95% confidence intervals. A) Spearman rank correlation between biodiversity and disease was not significantly associated with spatial extent. B) Pearson's skewness was negatively associated with spatial extent. C) Spearman rank correlation between biodiversity and disease was not significantly associated with spatial grain standardized by host mass. D) Pearson's skewness was not significantly associated with spatial grain standardized by host mass. Source data are provided as a Source Data file.

We also tested whether the effect of spatial scale on the shape of biodiversity-disease relationships was similarly sensitive to all three studies conducted at the largest spatial scale[1–3] by omitting all three studies and reanalyzing the data. Similar to omitting the Wood et al[1] study, omitting all three studies did not eliminate the significant effect of spatial extent on the monotonicity and direction of the relationship, but did eliminate the significant effect of grain on the monotonicity and direction of the relationship, and also eliminated the effect of spatial extent on the skew of the relationship. However, in contrast to removing the Wood et al[1] study alone, removing all three studies from analysis still resulted in a marginially significant effect of spatial grain on the skew of the relationship (Type III ANOVA $p = 0.055$; Supplementary Figure 14). Together these results highlight the sensitivity of these analyses to studies conducted at the largest spatial scale. These results therefore highlight the need for additional studies conducted at the largest spatial scales.

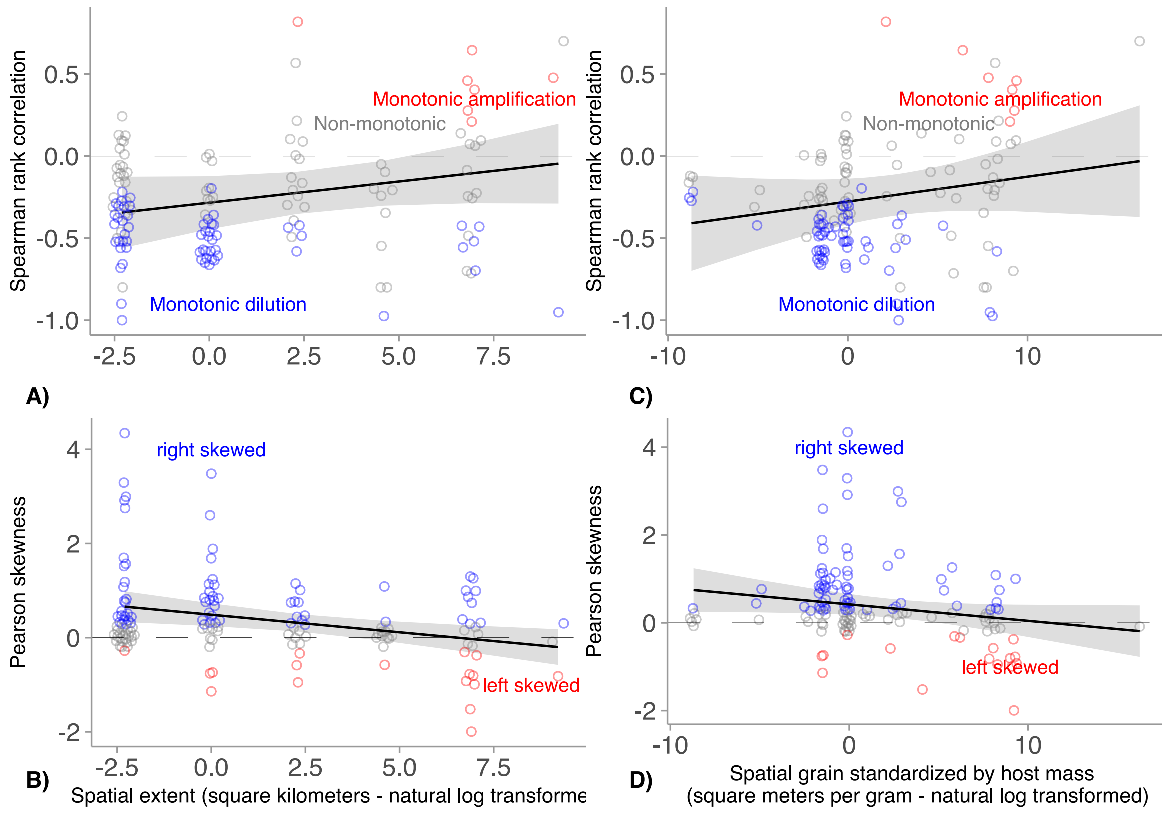

Supplementary Figure 14. Results of the analyses relating spatial scale to the shape of the biodiversity-disease relationship, omitting data from the study by Derne et al 2011[2], Nguyen et al 2016[3], and Wood et al 2017[1]. Points represent each published biodiversity-disease relationship, colored by their estimated shape (red = Monotonic amplification in panels A and C and left-skewed in panels B and D; blue = Monotonic dilution in panels A and C and right-skewed in panels B and D; grey = non-significant or non-monotonic in panels A and C non-skewed in panels B and D). Solid lines indicate the estimated fit of a multilevel random effects model, and grey ribbons indicate the 95% confidence intervals. A) Spearman rank correlation between biodiversity and disease was not significantly associated with spatial extent. B) Pearson's skewness was negatively associated with spatial extent. C) Spearman rank correlation between biodiversity and disease was not significantly associated with spatial grain standardized by host mass. D) Pearson's skewness was negatively associated with spatial grain standardized by host mass, but the effect was only marginally significant (Type III ANOVA $p = 0.055$). Source data are provided as a Source Data file.

Despite the sensitivity of these analyses to studies at the largest spatial scales, the effect of spatial scale on the shape and direction of the biodiversity-disease relationship remained qualitatively similar even when we excluded the three influential datasets at the largest spatial scales from our analysis. Even when we excluded the three influential datasets at the largest spatial scales from analysis, we still observed significant monotonic and negative relationships and significantly right-skewed relationships only occurring below threshold spatial scales. Specifically, after omitting the three studies conducted at the largest spatial extent, we still detected significantly monotonic negative and right-skewed biodiversity relationships only at spatial extents below <100km^2 (roughly the size of a small city), and at spatial grains below 100m^{2}g^{-1} (also roughly the size of a small city for a typical human parasite).

We next tested whether the effect of spatial scale on the shape of biodiversity-disease relationships was influenced by whether a study measured host richness, Shannon diversity, or Simpson's diversity, as well as whether a study measured disease prevalence or severity by fitting same two models, but including a two-way interaction between spatial scale and either the diversity metric or the disease metric. We found no evidence that the effect of spatial scale on biodiversity-disease relationships depended on either of these factors. However, we caution the interpretation of these results, as host richness was the only metric of host diversity that was measured across all spatial scales, no single metric of disease was measured across all spatial scales.

We tested whether several ecological factors could explain variation in the effect of spatial scale on the shape of biodiversity-disease relationships. Specifically, we tested whether the effect of spatial scale on biodiversity-disease relationships differed between (i) parasites that infect humans vs. wildlife, (ii) macro- vs. microparasites, (iii) parasites with complex vs. direct lifecycles, and (iv) observational vs. manipulative studies.

To test for context dependence in the spatial moderation of the biodiversity-disease relationship, we fit the same four models, but included a two-way interaction between spatial scale and four binary factors that might explain variation in the effects of scale on the biodiversity-disease relationship: parasite functional group (macroparasite vs. microparasite), parasite lifecycle (complex vs. direct), study design (manipulative vs. observational), and parasite type (infects humans vs. infects only wildlife).

We found no evidence that the effect of spatial scale on biodiversity-disease relationships depended on any of these factors (Supplementary Table 4). Thus, the effect of spatial scale on biodiversity-disease relationships was generally robust across all ecological contexts examined. However, we caution the interpretation of these results, as there was a high amount of multicollinearity in this analysis (Supplementary Figure 15, Supplementary Figure 16). Specifically, observational studies and studies of human pathogens both tended to occur at larger spatial scales than manipulative studies and studies of wildlife pathogens. Consequently, we cannot rule out the possibility that these results could change if future studies filled in these research gaps.

Supplementary Table 4. Models of ecological factors moderating the effect of spatial scale on biodiversity-disease relationships

	A) Spearman correlation coefficient			B) Pearson's skewness		
	**DF**	**F-value**	**p-value**	**DF**	**F-value**	**p-value**
Spatial extent	32.9	1.206	0.28	6.9	0.177	0.69
Human	39.5	0.116	0.74	47.4	0.100	0.75
Route	39.2	1.719	0.19	39.2	0.489	0.48
Macroparasite	29.0	1.055	0.31	9.4	0.140	0.71
Manipulative	38.4	0.262	0.61	12.7	0.004	0.94
Spatial extent × Human	34.4	0.073	0.79	26.7	0.076	0.78
Spatial extent × Route	38.2	0.269	0.61	52.6	0.374	0.54
Spatial extent × Macroparasite	30.5	0.576	0.45	18.4	0.285	0.59
Spatial extent × Manipulative	33.9	0.000	0.99	5.6	0.001	0.97
Spatial grain	28.2	1.216	0.28	160	0.252	0.62
Human	35.3	0.354	0.56	160	0.050	0.82
Route	34.8	0.296	0.59	160	0.670	0.41
Macroparasite	28.0	1.027	0.32	160	0.290	0.59
Manipulative	33.9	0.073	0.79	160	10.60	0.001
Spatial grain × Human	31.5	0.429	0.52	160	1.295	0.26
Spatial grain × Route	34.4	1.056	0.31	160	1.513	0.22
Spatial grain × Macroparasite	28.2	0.875	0.36	160	0.375	0.54
Spatial grain × Manipulative	32.3	0.124	0.72	160	0.344	0.56

Type III Analysis of Variance Table with Satterthwaite's method

DF: Denominator degrees of freedom

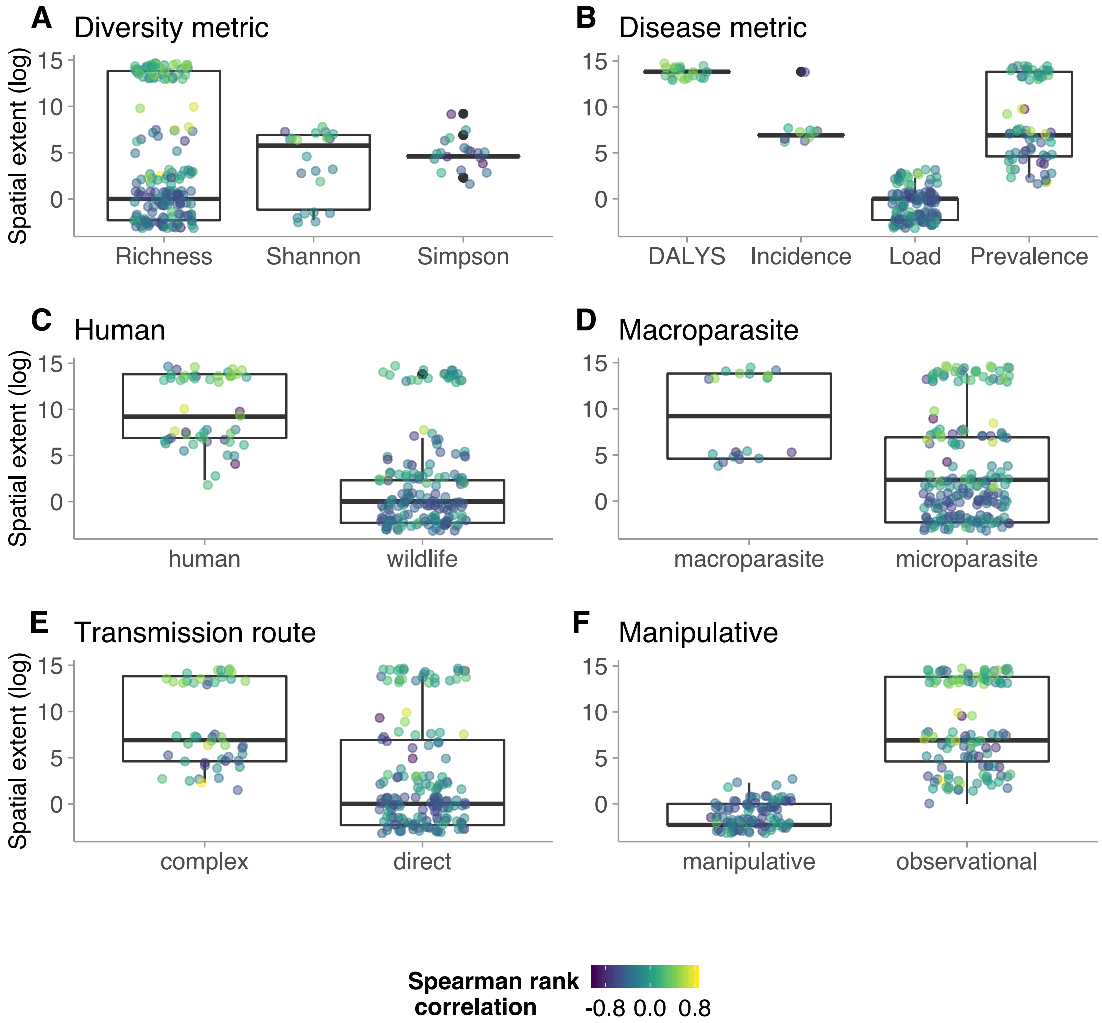

Supplementary Figure 15. Relationship between spatial extent and A) host diversity metric, B) disease metric, C) parasites that infect humans vs. wildlife, D) macro- vs. microparasites, E) parasites with complex vs. direct lifecycles, and F) observational vs. manipulative studies. Each point represents an individual study, colored by the Spearman rank correlation coefficient for the study, with numbers below zero (purple and dark-blue) indicating monotonic dilution and numbers above zero (light-green and yellow) indicating monotonic amplification effects. The box shows the first and third quartiles, the middle line shows the median, and the whiskers extend from the box to the largest and smallest values, no more than 1.5x the interquartile range. Source data are provided as a Source Data file**.**

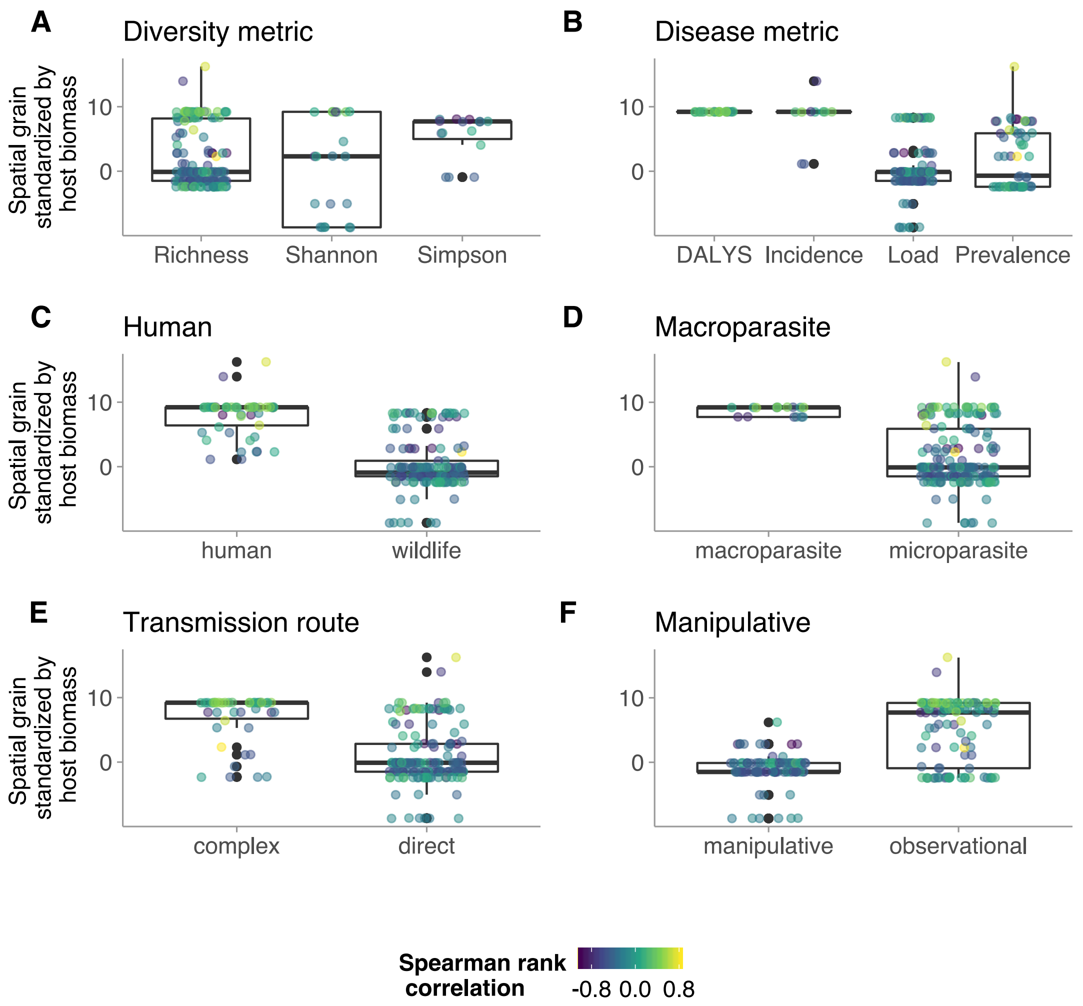

Supplementary Figure 16. Relationship between spatial grain and A) host diversity metric, B) disease metric, C) parasites that infect humans vs. wildlife, D) macro- vs. microparasites, E) parasites with complex vs. direct lifecycles, and F) observational vs. manipulative studies. Each point represents an individual study, colored by the Spearman rank correlation coefficient for the study, with numbers below zero (purple and dark-blue) indicating monotonic dilution and numbers above zero (light-green and yellow) indicating monotonic amplification effects. The box shows the first and third quartiles, the middle line shows the median, and the whiskers extend from the box to the largest and smallest values, no more than 1.5x the interquartile range. Source data are provided as a Source Data file**.**

Supplementary References

1. Wood, C. L., McInturff, A., Young, H. S., Kim, D. & Lafferty, K. D. Human infectious disease burdens decrease with urbanization but not with biodiversity. *Philos. Trans. R. Soc. B Biol. Sci.* **372**, 20160122 (2017).
2. Derne, B. T., Fearnley, E. J., Lau, C. L., Paynter, S. & Weinstein, P. Biodiversity and leptospirosis risk: A case of pathogen regulation? *Med. Hypotheses* **77**, 339–344 (2011).
3. Nguyen, D. *et al.* Fungal disease incidence along tree diversity gradients depends on latitude in European forests. *Ecol. Evol.* **6**, 2426–2438 (2016).
