# Supplementary material for: Measuring the shape of the biodiversity-disease relationship across systems reveals new findings and key gaps
Source: Nat Commun. 2019 Nov 6;10:5032. doi: 10.1038/s41467-019-13049-w (PMC6834853; doi:10.1038/s41467-019-13049-w)
Supplement: Supplementary file 3 — Reporting Summary [file 41467_2019_13049_MOESM3_ESM.pdf]

nature research

Corresponding author(s): *Double-blind peer review submissions: write DBPR and your manuscript number here instead of author names.*

Last updated by author(s): *YYYY-MM-DD*

Reporting Summary

Nature Research wishes to improve the reproducibility of the work that we publish. This form provides structure for consistency and transparency in reporting. For further information on Nature Research policies, see Authors & Referees and the Editorial Policy Checklist.

Statistics

For all statistical analyses, confirm that the following items are present in the figure legend, table legend, main text, or Methods section.

n/a	Confirmed	
☐	☒	The exact sample size (*n*) for each experimental group/condition, given as a discrete number and unit of measurement
☐	☒	A statement on whether measurements were taken from distinct samples or whether the same sample was measured repeatedly
☐	☒	The statistical test(s) used AND whether they are one- or two-sided<br>*Only common tests should be described solely by name; describe more complex techniques in the Methods section.*
☐	☒	A description of all covariates tested
☐	☒	A description of any assumptions or corrections, such as tests of normality and adjustment for multiple comparisons
☐	☒	A full description of the statistical parameters including central tendency (e.g. means) or other basic estimates (e.g. regression coefficient) AND variation (e.g. standard deviation) or associated estimates of uncertainty (e.g. confidence intervals)
☐	☒	For null hypothesis testing, the test statistic (e.g. F, t, r) with confidence intervals, effect sizes, degrees of freedom and P value noted<br>*Give P values as exact values whenever suitable.*
☒	☐	For Bayesian analysis, information on the choice of priors and Markov chain Monte Carlo settings
☐	☒	For hierarchical and complex designs, identification of the appropriate level for tests and full reporting of outcomes
☐	☒	Estimates of effect sizes (e.g. Cohen's *d*, Pearson's *r*), indicating how they were calculated

Our web collection on statistics for biologists contains articles on many of the points above.

Software and code

Policy information about availability of computer code

Data collection	We extracted data from text and tables manually and from figures using WebPlotDigitizer version 4.1, and recorded other data relating to the biology or methodology of each study.
Data analysis	All analyses were carried out in R version 3.5.0. We constructed multilevel random effects models using the lmer function in R packages lme4 and lmerTest. Pairwise comparisons were carried out using the R package lsmeans.

For manuscripts utilizing custom algorithms or software that are central to the research but not yet described in published literature, software must be made available to editors/reviewers. We strongly encourage code deposition in a community repository (e.g. GitHub). See the Nature Research guidelines for submitting code & software for further information.

Data

Policy information about availability of data

All manuscripts must include a data availability statement. This statement should provide the following information, where applicable:

- Accession codes, unique identifiers, or web links for publicly available datasets
- A list of figures that have associated raw data
- A description of any restrictions on data availability

The data and code supporting the results are archived on Figshare (DOI: 10.6084/m9.figshare.9784226).

Field-specific reporting

Please select the one below that is the best fit for your research. If you are not sure, read the appropriate sections before making your selection.

☐ Life sciences ☐ Behavioural & social sciences ☒ Ecological, evolutionary & environmental sciences

Ecological, evolutionary & environmental sciences study design

All studies must disclose on these points even when the disclosure is negative.

Study description	Criticism of observed relationships between host diversity and disease risk stems from three key concerns. First, most studies assume a linear, monotonic relationship between biodiversity and disease, though the actual shape is unknown. Second, most studies are conducted at a single spatial scale, though biotic interactions are often scale-dependent, and thus spatial scale might determine whether the relationship between biodiversity and disease is positive or negative. Third, most studies focus only on a small range of possible diversity levels, though the relationship between biodiversity and disease may change direction outside of this range. Resolving the debate surrounding the biodiversity-disease relationship therefore requires explicit consideration of multiple factors that are rarely included in any single study.<br><br>Here, we present an analysis of 205 biodiversity-disease relationships on 67 parasite species. We show that most biodiversity-disease relationships are non-linear, and non-monotonic, with biodiversity inhibiting disease at spatial extents less than 100 km2, though this effect weakens and might reverse as spatial scale increases
Research sample	This study comprised data from every published relationship between biodiversity and disease risk including two or more levels of host biodiversity.
Sampling strategy	This study aimed to analyze the shape of every published relationship between host diversity and the abundance of parasites. We updated the list of studies from Civitello et al. 2015 to include studies published between 2014 and 2018, by repeating their original search criteria. Specifically, we searched the Web of Science for several combinations of search terms: parasite, pathogen, diversity, richness, evenness, dilution effect, and amplification effect (the final search was conducted in June 2018). We identified additional papers by searching the literature cited sections of these articles and by searching Web of Science for all papers citing Civitello et al 2015, including those critical of the dilution effect hypothesis. We included observational and experimental studies in lab and field environments.<br><br>We only included studies that measured parasite abundance or prevalence at more than two host diversity levels. We included studies that reported infection prevalence, mean parasite load, density of infected vectors, or percent diseased tissue, because these quantities are the most relevant metrics of disease risk for microparasites, macroparasites, vector-borne parasites, and plant parasites, respectively.
Data collection	We extracted data from text and tables manually and from figures using WebPlotDigitizer version 4.1, and recorded other data relating to the biology or methodology of each study. For all studies, we recorded parasite and host taxa, type of parasite (infecting only wildlife or also infecting humans), focal host species, associated species (i.e., additional species whose presence may dilute or amplify parasite abundance, operationally defined as “potential diluters”), the diversity (e.g., richness) in the treatments (or in the field survey), parasite functional group (macroparasite vs. microparasite), parasite lifecycle (complex vs. direct), and study design (manipulative vs. observational). Spatial extent was quantified as the area (expressed in square kilometers) over which all biodiversity estimates were compared in a given study. Studies rarely provided an exact value for spatial extent. Because a value for spatial extent was rarely provided, and spatial extents varied by six orders of magnitude, we estimated the extent of each survey to the nearest order of magnitude rather than attempting to assign a specific spatial extent for each study. For example, we assigned studies a value of 0.1 if the extent was less than 1 km2, and a value of 1 if the extent was greater than 1 km2, but less than 10 km2, etc.
Timing and spatial scale	The final search on Web of Science was conducted in June 2018
Data exclusions	We omitted studies with fewer than four unique measures of host diversity for Spearman rank correlations and unconstrained splines and fewer than three unique measures of host diversity for CQ splines. Twenty-nine of the unconstrained splines (n=205) and 39 of the CQ splines (n=217) showed no relationship between biodiversity and disease (e.g., a fit with a slope of zero), resulting in no estimate of Pearson’s skewness. This resulted in 205 estimates of [illegible]176 estimates of skew from unconstrained splines, and 178 estimates of skew from CQ splines.
Reproducibility	This study was not a new experiment, but rather a large analysis of prior published data.
Randomization	This study was not experimental.
Blinding	This was not an experimental study.

Did the study involve field work? ☐ Yes ☒ No

Reporting for specific materials, systems and methods

We require information from authors about some types of materials, experimental systems and methods used in many studies. Here, indicate whether each material, system or method listed is relevant to your study. If you are not sure if a list item applies to your research, read the appropriate section before selecting a response.

Materials & experimental systems

n/a	Involved in the study
☒	☐ Antibodies
☒	☐ Eukaryotic cell lines
☒	☐ Palaeontology
☒	☐ Animals and other organisms
☒	☐ Human research participants
☒	☐ Clinical data

Methods

n/a	Involved in the study
☒	☐ ChIP-seq
☒	☐ Flow cytometry
☒	☐ MRI-based neuroimaging
