# Supplementary material for: Measuring the shape of the biodiversity-disease relationship across systems reveals new findings and key gaps
Source: Nat Commun. 2019 Nov 6;10:5032. doi: 10.1038/s41467-019-13049-w (PMC6834853; doi:10.1038/s41467-019-13049-w)
Supplement: Supplementary file 4 — Description of Additional Supplementary Files [file 41467_2019_13049_MOESM4_ESM.pdf]

Description of Additional Supplementary Files

File Name: Supplementary Data 1
Description: Akaike information criterion (AIC), Bayesian information criterion (BIC), and R^2 for each model fit to each biodiversity-disease relationship
